# Case Report: Hypoglycemia Due to a Novel Activating Glucokinase Variant in an Adult – a Molecular Approach

**DOI:** 10.3389/fendo.2022.842937

**Published:** 2022-03-17

**Authors:** Anojian Koneshamoorthy, Dilan Seniveratne-Epa, Genevieve Calder, Matthew Sawyer, Thomas W. H. Kay, Stephen Farrell, Thomas Loudovaris, Lina Mariana, Davis McCarthy, Ruqian Lyu, Xin Liu, Peter Thorn, Jason Tong, Lit Kim Chin, Margaret Zacharin, Alison Trainer, Shelby Taylor, Richard J. MacIsaac, Nirupa Sachithanandan, Helen E. Thomas, Balasubramanian Krishnamurthy

**Affiliations:** ^1^ Department of Endocrinology and Diabetes, St. Vincent’s Hospital, Melbourne, VIC, Australia; ^2^ St. Vincent’s Institute of Medical Research, Melbourne, VIC, Australia; ^3^ Department of Medicine, St. Vincent’s Hospital, Melbourne, VIC, Australia; ^4^ Department of Surgery, St. Vincent’s Hospital, Melbourne, VIC, Australia; ^5^ Melbourne Integrative Genomics, Faculty of Science, University of Melbourne, Melbourne, VIC, Australia; ^6^ Charles Perkins Centre, School of Medical Sciences, University of Sydney, Sydney, NSW, Australia; ^7^ Department of Diabetes and Endocrinology, Royal Children’s Hospital, Melbourne, VIC, Australia; ^8^ Department of Genomic Medicine, Royal Melbourne Hospital, Melbourne, VIC, Australia

**Keywords:** hypoglycemia, glucokinase, congenital hyperinsulinism, hyperplastic islets, MODY

## Abstract

We present a case of an obese 22-year-old man with activating *GCK* variant who had neonatal hypoglycemia, re-emerging with hypoglycemia later in life. We investigated him for asymptomatic hypoglycemia with a family history of hypoglycemia. Genetic testing yielded a novel *GCK* missense class 3 variant that was subsequently found in his mother, sister and nephew and reclassified as a class 4 likely pathogenic variant. Glucokinase enables phosphorylation of glucose, the rate-limiting step of glycolysis in the liver and pancreatic β cells. It plays a crucial role in the regulation of insulin secretion. Inactivating variants in *GCK* cause hyperglycemia and activating variants cause hypoglycemia. Spleen-preserving distal pancreatectomy revealed diffuse hyperplastic islets, nuclear pleomorphism and periductular islets. Glucose stimulated insulin secretion revealed increased insulin secretion in response to glucose. Cytoplasmic calcium, which triggers exocytosis of insulin-containing granules, revealed normal basal but increased glucose-stimulated level. Unbiased gene expression analysis using 10X single cell sequencing revealed upregulated *INS* and *CKB* genes and downregulated *DLK1* and *NPY* genes in β-cells. Further studies are required to see if alteration in expression of these genes plays a role in the metabolic and histological phenotype associated with glucokinase pathogenic variant. There were more large islets in the patient’s pancreas than in control subjects but there was no difference in the proportion of β cells in the islets. His hypoglycemia was persistent after pancreatectomy, was refractory to diazoxide and improved with pasireotide. This case highlights the variable phenotype of *GCK* mutations. In-depth molecular analyses in the islets have revealed possible mechanisms for hyperplastic islets and insulin hypersecretion.

## Introduction

Glucokinase (also known as hexokinase IV, encoded by the *GCK* gene) enables phosphorylation of glucose, the rate-limiting step of glycolysis in the liver and pancreatic β cells (1). It plays a crucial role in the regulation of insulin secretion (2). GCK is the primary glucose sensor as small fluctuations in its activity alter glucose-stimulated insulin secretion from pancreatic β cells (3). GCK’s midpoint of glucose responsiveness is 7 mM (as compared to ~0.2 mmol/L for homologous isozymes hexokinases I-III), which closely matches physiological, circulatory glucose concentrations (3). Unlike the other hexokinases, GCK is not susceptible to feedback inhibition by physiological concentrations of its product, glucose 6-phosphate (3). The importance of precise control over GCK activity is emphasized by disease phenotypes resulting from variants in the human GCK locus (3). Inactivating variants in *GCK* cause hyperglycemia (4) and activating variants cause hypoglycemia (5).

Here we report detailed clinical, functional, and molecular analysis of a man who presented with hypoglycemia due to an activating variant in *GCK*. The patient underwent spleen-preserving distal pancreatectomy with a curative intent for his hypoglycemia. Histology revealed diffuse larger islets, nuclear pleomorphism and periductular islets. Genetic testing revealed a novel activating *GCK* variant that was subsequently also found in his mother, sister and nephew. Islets were isolated from his pancreas and subjected to functional analysis and single cell RNA sequencing to reveal possible mechanisms for the metabolic effects of the activating glucokinase variant and associated morphological changes in islets.

### Clinical Case

A 22-year-old man was referred for evaluation of asymptomatic hypoglycemia (plasma glucose 2.4 mmol/L) which was found incidentally during investigation for obesity (BMI 49.1 kg/m^2^). He has no history of bariatric surgery, liver or kidney disease or excess alcohol consumption. His mother had a pancreatectomy (of unknown extent) at age 6 for hypoglycemic seizures. She developed diabetes mellitus in her fifth decade and she is on insulin. There was no history of glucose homeostasis related issues in his father. His sister was asymptomatic when the patient was first evaluated, but she had hypoglycemia when she was monitoring her glucose after diagnosis of gestational diabetes mellitus (described in detail later).

**Figure 1:**
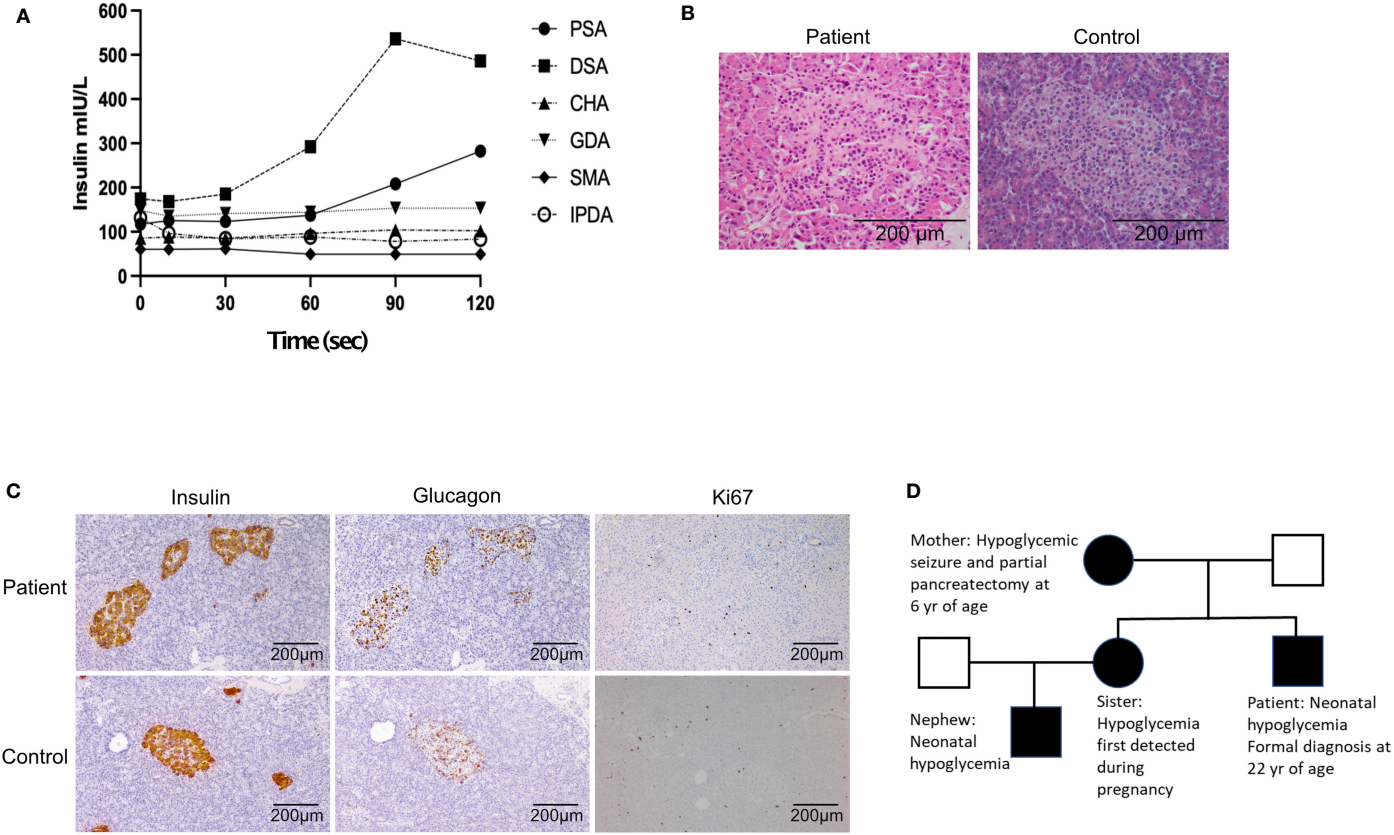
**(A)** Selective arterial calcium stimulation test: Insulin level obtained during a selective arterial calcium stimulation test. Increased hepatic vein insulin when calcium gluconate (0.025 mEq Ca^2+^/kg diluted to a 5-mL in normal saline, given as rapid bolus) was injected into the distal splenic artery suggested a focal abnormal insulin production in the region the body and tail of the pancreas. PSA, proximal splenic artery; DSA, distal splenic artery; CHA, common hepatic artery; GDA, gastro duodenal artery; SMA, superior mesenteric artery; IPDA, inferior pancreatico duodenal artery. **(B)** Hematoxylin and eosin high power (x200) - variation in size of islet cell nuclei. **(C)** Representative histology of patient (top panels) and control (bottom panels) pancreas sections stained with antibodies recognizing insulin, glucagon and Ki67. **(D)** Pedigree of patient. Black represents individuals with confirmed GCK mutation.

His initial investigation is shown in **Table 1**. A prolonged fast demonstrated endogenous hyperinsulinemic hypoglycemia (**Table 2**). The test was terminated at 10 hours of fasting when the patient’s blood glucose was < 2.5mM. Imaging of the pancreas with triple phase computed tomography (CT), magnetic resonance imaging (MRI), endoscopic ultrasound, ^68^Ga-DOTA-octreotate PET/CT (Dotatate scan, targeting somatostatin receptor subtype 2) and ^68^Ga-DOTA-exendin-4 PET/CT (GLP-1R scan, targeting glucagon-like peptide-1 receptor) did not reveal a pancreatic lesion (not shown). A selective arterial calcium stimulation test suggested focal abnormal insulin production in the body and tail region (distal splenic artery territory) of the pancreas (**Figure 1A**). Hence he underwent spleen-preserving distal pancreatectomy with resection of the body and tail of the pancreas to the left of the superior mesenteric vein–portal vein confluence with a curative intent for his hypoglycemia. At the time of surgery, we were unaware of the genetic cause for hypoglycemia in the patient. An intraoperative ultrasound did not reveal any pancreatic lesions. Histopathology revealed mildly hyperplastic islets, nuclear pleomorphism and periductular islets (**Figures 1B, C** **)**. There was no increase in Ki67 expressing islet cells in the patient as compared to control islets (**Figure 1C**). The distal pancreatectomy, however, did not correct hypoglycemia, as demonstrated with continuous glucose monitoring (CGM). During the diagnostic evaluation and postoperatively, he had no improvement in his hypoglycemia with continuous glucose monitoring revealing about 50-70% of time below 3.9 mm, with verapamil (80 mg oral, twice daily), a calcium channel blocker, and diazoxide (100 mg oral, three times a day, he could not tolerate a higher dose as he gained 14 kg of weight in 6 weeks with diazoxide), which inhibits insulin release by opening β-cell ATP-sensitive potassium channels. Similarly, octreotide (200 mcg sc three times a day), a somatostatin analogue which inhibits insulin release by activating somatostatin receptors 2 and 5 did not improve his hypoglycemia. There was improvement in hypoglycemia with pasireotide (900 mcg sc twice a day), another somatostatin analogue that activates somatostatin receptors 1, 2, 3 and 5 and has much higher binding affinity for somatostatin receptors 1, 3 and 5 than octreotide. While there was decrease in time below 3.9 mM to 20%, the time above 7.8 mM increased to about 30% with pasereotide (**Supplementary Figures 1**, **2**). There was little change in his insulin or c-peptide values after the surgery. His weight decreased from 141.0 kg before surgery to 123 kg after surgery. His plasma triglyceride was normal (1.9 mM).

**Table 1:** Initial biochemical parameters*.

Test	Value	Reference range
**Plasma Glucose**	2.3	3.0 – 7.7 mmol/L
**C-Peptide**	904	268-1275 pmol/L^1^
**Insulin**	17.8	3- 25 mU/L^1^
**Pro-Insulin**	37.1	<13 pmol/L
**Betahydroxybutyrate**	<0.01	0 – 0.61 mmol/L^1,2^
**HbA1c**	3.6	4.0-6.0%
**Sulphonylurea screen**	Negative	
**Insulin antibodies**	Negative	
**Thyroid stimulating hormone**	3.65	0.5-4.7 mIU/L
**Free T4**	13.9	11.5-22.7 pmol/L
**Cortisol**	265	100-535 nmol/L
**Insulin-like growth factor-1**	18	12-42 nmol/L
**Human growth hormone**	<0.1	0-1.7 ug/L
**Amino acids**	Non diagnostic profile	
**Lactate**	1.5	0.5-2.2 mmol/L
**Ammonia**	55	16-53 μmol/L

*The patient blood sample was drawn in non-fasted state at 11.30 am.

^1^Reference range for normoglycemia in fasted state.

^2^Betahydroxybutyrate in fasted, hypoglycaemic state would be 2-4 mmol/L.

**Table 2:** Prolonged fasting test.

Time	Glucose (mmol/L)	Insulin (mU/L)	C-peptide (pmol/L)	Pro insulin (pmol/L)	B-hydroxy butyrate (mmol/L)	Comments
1200 (+0 hrs)	3.8					Fasting from 1200
1800 (+6 hrs)	2.5	24	126	>100	0.08	
2000 (+8 hrs)	2.3	7	94	37.1	0.05	
2200 (+10 hrs)	1.9	7	359	–	0.05	No symptoms Given glucagon 1mg
2210 (+1010 hrs)	6.1					
2220 (+1020 hrs)	5.2					

The index case’s pediatrician was later identified during the discussion on management of his nephew. Discussion with his pediatrician revealed that the index case’s birth weight was 4.2 kg. He had a history of severe, recurrent, neonatal hyperinsulinemic hypoglycemia, treated effectively with diazoxide. His hypoglycemia improved and he was weaned off diazoxide by the age of 3 years. He developed obesity within the first 2 years of life. He was lost to follow up after 3 years.

### Family Pedigree

The patient’s sister developed gestational diabetes based on oral glucose tolerance test with 75 g of glucose during the second trimester. During the pregnancy, home glucose monitoring showed fasting glucose levels between 3.5 and 4.5 mmol/L along with post-prandial hypoglycemia despite not being on insulin. She delivered a male baby with birth weight of 3026g (25^th^ percentile). He developed hypoglycemia with plasma glucose of 1.3 mmol/L at 6 hours of life. The infant had recurrent hypoglycemia, requiring intravenous dextrose and glucagon. He was responsive to diazoxide and was discharged from hospital at 5 weeks of age with regular feeding intervals and a diazoxide regimen of 12mg/kg/day. The family pedigree is shown in **Figure 1D**.

## Results

### Genetic Analysis Reveals an Activating Glucokinase Variant

Genetic testing by analysis of the genes known to cause hypoglycemia by targeted next generation sequencing was performed in the patient and his mother (Exeter laboratory). This revealed a novel heterozygous glucokinase (*GCK*) variant (c.269A>C p.(Lys90Thr), which was predicted to be a pathological mutation using tools including Sorting Intolerant From Tolerant (SIFT), Grantham Variation and Grantham Deviation (Align-GVGD) and Polymorphism Phenotyping v2 (PolyPhen-2). Based on international ACMG guidelines, this variant was originally classified as a class 3 variant of uncertain significance. Segregation analysis for the variant in the index case’s sister and her son revealed the same *GCK* c.269A>C p.(Lys90Thr) variant and the variant was re-classified as a class 4 likely pathogenic variant. This case report highlights the importance of awareness of the possibility of genetic forms of hyperinsulinism in adults with hypoglycemia especially when imaging is uninformative and the need to search for clues in the past medical and family histories.

### Pancreatic Islet Analysis

#### Increased Intracellular Calcium in Response to Glucose

**Figure 2:**
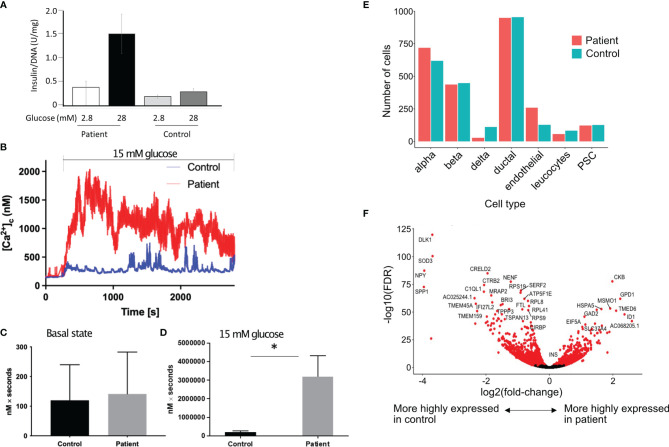
Increased insulin secretion by the islets. **(A)** *In vitro* glucose stimulated insulin secretion of isolated islets cells. Data show mean ± SD of 3 replicates. **(B)** Increase in cytoplasmic calcium level (which triggers degranulation of insulin granules) in response to 15 mM glucose in patient’s islets (red) as compared to control islets (blue). **(C, D)** Area under the curve (expressed as nM x seconds) of cytoplasmic calcium in islets in the basal state **(C)** and during stimulation with 15 mM of glucose **(D).** Data show mean ± SD of 3 replicates. *p=0.0289, 2 tailed unpaired t test. **(E)** Proportion of different cell types in the islets from the patient as compared to a single control using 10X single cell sequencing. **(F)** Volcano plot showing differentially expressed genes in the β cells of patient’s islets as compared to control islets.

Islets were isolated from a part of the removed pancreas for functional studies. Glucose stimulated insulin secretion (GSIS) was higher in patient’s islets as compared to control islets (**Figure 2A**). Cytoplasmic calcium ([Ca^2+^]_c_), which triggers exocytosis of insulin-containing granules and insulin secretion, was measured in islets from the patient, and control islets from a normal donor by Fura-2 live-cell calcium imaging. Basal [Ca2+]_c_ (*0–180 s*) did not significantly differ between patient and control islets (**Figures 2B, C** **)**. [Ca^2+^]_c_ was significantly elevated in the patient’s islets in response to 15 mM glucose (*181–2700 s*), compared to the control (**Figures 2B, D** **)**. In normal islets, intracellular concentrations of calcium are maintained at approximately 100 nM in unstimulated islets and increase to approximately 500 nM following stimulation with secretagogues (6). It is possible that the high cytosolic calcium in response to glucose could be due to the patient’s obesity.

#### Single Cell Transcriptomics Reveals Increased INS Expression and Downregulation of Genes That Control β-Cell Maturation

To investigate the molecular basis for the altered metabolism and larger islet noted in histology, we examined whole transcriptome gene expression changes using 10X single cell sequencing from the patient’s islets compared with control islets. There were 1,191 genes significantly upregulated and 1,636 genes significantly downregulated in the patient’s b cells compared with the control b cells (FDR < 0.01 using edgeR’s quasi-likelihood F-test). There was no difference in the proportion of islet cell types, including β cells, in the islets from the patient as compared to control islets (**Figure 2E** **)**. There were more islets per gram of pancreatic tissue in the patient. We isolated 13,292 islet equivalent (IEQ) per gram of digested pancreas from the patient’s pancreas as compared to 5,209 IEQ ± 2,125 IEQ in controls (mean ± SD of 20 donors aged between 18-26 years of age). The islets were also larger; large islets (>250µm) from the patient contributed 52% of the total islet mass compared to mean ± SD of 29.43 ± 10.53% (range 15-46.3%) from 10 nondiabetic donors between 18 and 26 years of age. The control subjects were matched to the patient for age but the average BMI of the control donors was 27 ± 4.8 kg/m^2^. If we only consider control subjects with BMI>27 (n=5), the mean contribution to IEQ by islet >250 µm is 29.48 ± 11.89%. While large islets in patients with activating glucokinase mutation have been described previously (7), it is likely that the patient’s obesity contributed to the greater islet size. Single cell RNA sequencing revealed that the patient’s β cells expressed significantly more *INS* (insulin) than the control β cells. Creatine kinase B (CKB) reversibly catalyzes the transfer of phosphate between ATP and creatine or between phospho-creatine and ADP. *CKB* was highly expressed in the patient’s β cells (**Figure 2F**).

To investigate the underlying mechanisms for larger islets in the patient, we checked if the islets are maintained in a more proliferative stage, similar to neonatal islets. *DLK1* and *NPY* are highly expressed in neonatal islets and both function in β cells to inhibit the switch from immature proliferating cells to a mature differentiated state (8, 9). We found both *DLK1* and *NPY* to be significantly downregulated in the patient’s β cells (**Figure 2F**). In addition, expression of genes involved in glycolysis, cell cycle determination or β cell differentiation was not different in the patient’s β cells as compared to control β cells (data not shown). As there was increased insulin expression in the patient, we checked if autocrine insulin receptor (IR) signalling in β cells or the high local insulin concentration in islets could bind to IGF-1 to induce β cell proliferation (10–13). We did not find a difference in *IR*, *IGF-1R*, the downstream *IRS-1* and *IRS-2* or the negative regulator *KIAA1324* (Inceptor) gene expression (14) in the patient’s β cells as compared to the control β cells. The level of hexokinase subtypes was low in the β cells in patient and control sample and there was no difference in expression levels between the patient and control β cells.

## Discussion

We report a family with hyperinsulinemic hypoglycemia due to a novel activating *GCK* variant. The metabolic effects of activating glucokinase are due to changes in the threshold for insulin release. In-depth molecular analysis of β cells revealed genes that possibly contribute to the process of lowering the threshold for insulin release and associated morphological changes in pancreatic islets in activating glucokinase variant. His massive obesity may also have contributed to the morphological changes in the islets and increased calcium responses to glucose. We noted increased glucose stimulated insulin secretion in the patient’s β cells. *INS* and *CKB* were highly expressed while *DLK1* and *NPY* were prominently downregulated in the patient’s β cells.

GCK has been detected in the pancreas, liver, gut, and brain and is shown to play a key role in the regulation of carbohydrate metabolism. It acts as a glucose sensor in pancreatic β cells and promotes the synthesis of glycogen and triglycerides in the liver (15, 16). A patient with *GCK* c.269A>G, p.(Lys90Arg) variant, which is similar to the variant in our patient was described recently (17). Adults who are identified with activating *GCK* variants are usually diagnosed with hyperinsulinemic hypoglycemia as part of family screening following an identified neonate (18). Despite carrying the same *GCK* variant, the clinical presentation in this family varied significantly between affected relatives. Several studies have noted variation in clinical presentation within families with activating glucokinase variants (5, 19). Although speculative, one possible explanation for this phenomenon is individual differences in the way liver-specific glucokinase regulatory protein (GKRP), the regulator of GCK activity in the liver, mediates GCK inhibition in individuals.

Functional and transcriptomic analysis of islets isolated from the patient and a control pancreas suggested an increase in β-cell function rather than increased β-cell proliferation. While the number of islets per gram of pancreatic tissue was more in the patient than in control subjects, single cell analysis showed no difference in the proportion of β cells in the patient compared to control islets. However, in addition to the larger islets, many single chromogranin A positive cells were seen throughout the pancreas (data not shown), a phenomenon not usual in normal adult human pancreas. Thus, while the proportion of β cells was not different in the isolated islets, overall, the number of β cells in the patient’s pancreas was likely higher because these single β cells would have been lost during islet isolation.

Glucose stimulated insulin secretion (GSIS) was increased in the patient’s islets. *CKB* and *INS* were highly expressed in the patient’s β cells and intracellular Ca^2+^ levels were increased. *CKB* is important for regeneration of ATP and thus for GSIS. Insulin potentiates GSIS *in vivo* in healthy humans (20). *In vitro* stimulation of β cells with insulin leads to an increase in intracellular Ca^2+^ levels and insulin secretion (21) (22),. While glucose is the predominant insulin secretagogue, insulin mediated increase in intracellular Ca^2+^ may also amplify insulin secretion (21, 22),. Increased GSIS by the patient’s β cells may also be explained by low expression of *NPY*. *NPY* regulates insulin release from β cells by reducing cAMP levels. Knockdown of *Npy* expression in neonatal mouse islets enhances insulin secretion in response to high glucose (8). Thus, increased expression of *INS* and *CKB* and decreased expression of *NPY* potentially contribute to the lower threshold for glucose stimulated insulin release.

*DLK1* was reduced in the patient’s β cells. *DLK1* is highly expressed in normal β cells and may regulate local insulin action (23, 24), by inhibiting insulin signaling (25). *Dlk1* knockout mice are insulin sensitive and have increased β cell mass, while transgenic mice overexpressing DLK1 under control of the collagen promoter have impaired insulin sensitivity (25). Decreased DLK1 may allow unrestrained insulin signaling leading to β-cell hypertrophy. However, mice with β-cell specific DKL1 deficiency do not have larger islets (26). Interestingly, DLK1 mutation has been linked to central precocious puberty and obesity in humans (27). Accelerated weight gain was also noted in *Dlk1* null mice (28). It is not clear how a gain of function in glucokinase modulates the expression of *DLK1*.

## Conclusion

Our study highlights that genetic hyperinsulinemic hypoglycemia should be considered as a differential diagnosis in adults with hypoglycemia, especially when imaging is uninformative. Single cell transcriptome analysis revealed changes in gene expression in the islets with activating glucokinase variant. Further studies are required to see if alteration in gene expression plays a role in the metabolic and histological phenotype associated with the pathogenic glucokinase variant.

## Materials and Methods

### Human Islets Isolation and Single Cell RNA Sequencing

Pancreas following distal pancreatectomy was obtained, with informed consent, following research approval from the Human Research Ethics Committee at St. Vincent’s Hospital Melbourne (HREC-011-04). Human islets were isolated from the pancreas of the index case using standard procedures (29). We also isolated islet from a control subject whose pancreas was donated at the time we isolated the patient’s islets. We quantified the isolated islets using the standard islet equivalency (IEQ) method for islet volume measurements used in islet transplantation. One IEQ is equal to a single spherical islet of 150 μm in diameter, estimated microscopically after dithizone staining (30). Islets were dispersed with accutase then rested in Connaught Medical Research Laboratories (CMRL) 1066 medium (Invitrogen) supplemented with 4% human serum albumin, 100 U/ml penicillin, 100 mg/ml streptomycin and 2 mM L-glutamine (complete CMRL), in a 37°C, 5% CO2 humidified incubator for 2 hours. Cells were processed for single cell RNAseq using the Chromium 10x genomics platform and sequenced using the Illumina NextSeq platform at the Australian Genome Research Facility (Melbourne, Australia).

### Data Preprocessing

Patient and control subject’s single-cell libraries were multiplexed and sequenced across 4 lanes. The raw sequencing files (.BCL) were first converted into fastq files using “cellranger mkfastq” (v3.02), were then processed using “cellranger count” (3.0.2) pipeline to align sequencing reads in fastq files to reference genome GRCh38 and generate the raw gene count matrices for single cells in each sample.

### Normalisation and Clustering

The cells from two samples (patient and control islets) were pooled together for clustering and cell type identification. The filtered feature matrices from two samples (output by cellranger count) were imported into R (3.6) as a Single Cell Experiment (31) object (9,673 cells in total). Normalised gene expression was obtained by applying sctransform (v0.2.0) (32). Principle Component Analysis was performed on normalised data using the top 3,000 highly variable genes selected by their residual variance from fitting sctransform and the top 10 PCs were used for clustering using the SNN-based (shared-nearest-neighbour) clustering function from scran (v1.12.1) (33). The highly expressed genes from each cluster were used as cluster markers and compared to cell type markers found from the literature (34) for cell type identification. Cell type composition plots were generated by ggplot2 (3.1.1).

### Differential Gene Expression

Differential gene expression analysis was conducted within identified cell type groups contrasting the cells from case versus from control sample using edgeR(3.26.4) (35) based on a quasi-likelihood F-test (30) and including the cellular detection rate (the fraction of detected genes per cell) as a covariate (36). The genes had log10_total_counts lower than 1.5 were excluded. The significance determined using an FDR (false discovery rate) threshold of 0.01. Note that with only one patient sample and one control sample processed in separate batches, differential expression p-values should be taken as indicative only and will not be calibrated at the nominal significance level.

### Glucose Stimulated Insulin Secretion

Islets (200 IEQ) were pre-incubated for 1 hour in HEPES-buffered-KREBS buffer containing 0.1% BSA and 2.8 mmol/L D-glucose in triplicate. Islets were then incubated at 37°C for another 1 hour in KREBS buffer containing either 2.8 mmol/L or 28 mmol/L D-glucose. Culture medium was collected, and insulin secretion was measured by ELISA (Mercodia, Uppsala, Sweden) and values normalized to extracted islet DNA.

### Cytoplasmic Calcium Ion Response to Glucose

The [Ca^2+^]_c_ in islets was determined by live cytosolic calcium imaging as previously described (37, 38).

### Immununohistochemistry

Pancreatic specimens were fixed in 10% neutral buffered formalin. Antibodies for immunohistochemistry were guinea pig anti-insulin (Dako, Carpinteria, CA, USA), mouse anti-glucagon (Sigma-Aldrich, St. Louis, MO), mouse anti human Ki-67 (Dako), mouse anti human chromogranin A (AbDSerotec, Raleigh, NC), rabbit anti guinea pig HRP (Dako), rabbit anti-mouse HRP (Dako). Sections (5 µm) were stained using a Dako autostainer, detected with a peroxidase substrate containing 3,3-diaminobenzidine (brown) (Dako) and counterstained with haematoxylin.

### Genetic Testing

Whole blood from the patient and his mother was sent to the Exeter Clinical Laboratory for genetic testing for analysis of the coding regions and exon/intron boundaries of the *KCNJ11, ABCC8, AKT2, GLUD1, GCK, GPC3, HADH, HNF4A, INSR, KDM6A, KMT2D, SLC16A1, CACNA1D, PMM2, TRMT10A* and *HNF1A* genes by targeted next generation sequencing (Agilent custom capture v5.3/Illumina NextSeq500).

## Data Availability Statement

The single cell RNA seq data have been uploaded to GEO (https://www.ncbi.nlm.nih.gov/geo/) with the accession number GSE193693.

## Ethics Statement

Human islet studies were approved by the St Vincent’s Hospital Human Research Ethics Committee (HREC-011-04). The patients/participants provided their written informed consent to participate in this study.

## Author Contributions

Conceptualization, BK, TK, PT, and HT. Methodology, TL, LM, RL, XL, and JT Formal analysis, BK, HT, RL, and DM. Investigation, AK, DS, GC, MS, SF, LC, MZ, AT, RM, and NS. Writing—original draft preparation, AK and BK. Writing—review and editing, TK, PT, MZ, HT, and BK. Funding acquisition, TK and HT. All authors have read and agreed to the published version of the manuscript.

## Funding

This research was funded by The National Health and Medical Research Council of Australia, Grant number 1150425.

## Conflict of Interest

The authors declare that the research was conducted in the absence of any commercial or financial relationships that could be construed as a potential conflict of interest.

## Publisher’s Note

All claims expressed in this article are solely those of the authors and do not necessarily represent those of their affiliated organizations, or those of the publisher, the editors and the reviewers. Any product that may be evaluated in this article, or claim that may be made by its manufacturer, is not guaranteed or endorsed by the publisher.
