# Supplementary material for: Case Report: Hypoglycemia Due to a Novel Activating Glucokinase Variant in an Adult – a Molecular Approach
Source: Front Endocrinol (Lausanne). 2022 Mar 17;13:842937. doi: 10.3389/fendo.2022.842937 (PMC8969599; doi:10.3389/fendo.2022.842937)
Supplement: Supplementary file 1 [file Presentation_1.pptx]

## Slide 1
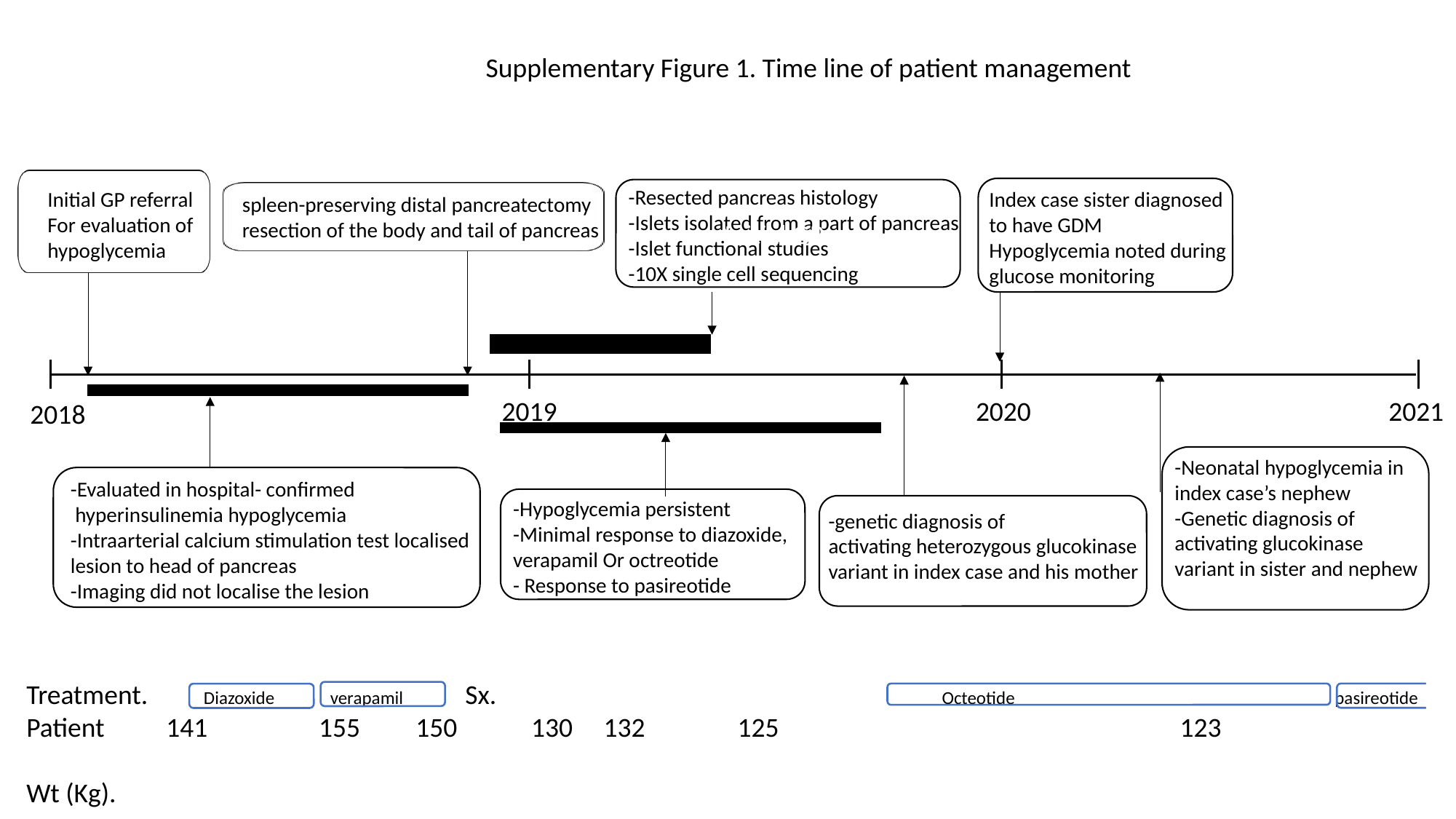

Supplementary Figure 1. Time line of patient management
l GPm
-Resected pancreas histology
-Islets isolated from a part of pancreas
-Islet functional studies
-10X single cell sequencing
Initial GPm
Initial GP referral
For evaluation of
hypoglycemia
Index case sister diagnosed to have GDM
Hypoglycemia noted during glucose monitoring
spleen-preserving distal pancreatectomy
resection of the body and tail of pancreas
2019
2020
2021
Initial GPm
-Neonatal hypoglycemia in index case’s nephew
-Genetic diagnosis of activating glucokinase
variant in sister and nephew
Initial GPm
-Evaluated in hospital- confirmed
 hyperinsulinemia hypoglycemia
-Intraarterial calcium stimulation test localised
lesion to head of pancreas
-Imaging did not localise the lesion
Initial GPm
-Hypoglycemia persistent
-Minimal response to diazoxide,
verapamil Or octreotide
- Response to pasireotide
Initial GPm
-genetic diagnosis of
activating heterozygous glucokinase
variant in index case and his mother
2018
Treatment. Diazoxide verapamil Sx. Octeotide pasireotide
Patient 141 155 150 130 132 125 123
Wt (Kg).

## Slide 2
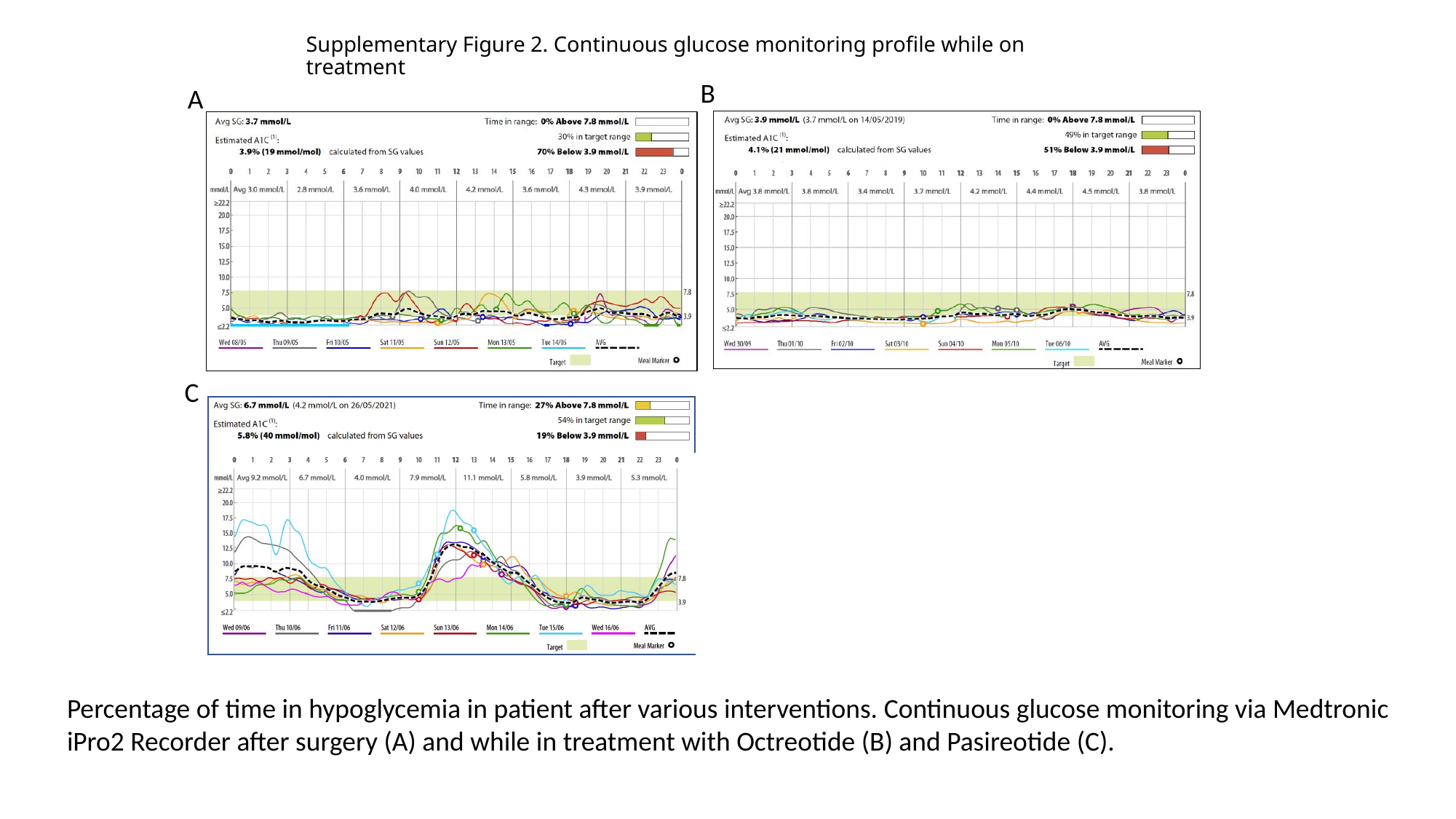

# Supplementary Figure 2. Continuous glucose monitoring profile while on treatment
B
A
C
Percentage of time in hypoglycemia in patient after various interventions. Continuous glucose monitoring via Medtronic iPro2 Recorder after surgery (A) and while in treatment with Octreotide (B) and Pasireotide (C).
